# Design of Human-Inspired Feet to Enhance the Performance of the Humanoid Robot Mithra

**DOI:** 10.3390/biomimetics10100675

**Published:** 2025-10-07

**Authors:** Spencer Brewster, Paul J. Rullkoetter, Siavash Rezazadeh

**Affiliations:** Department of Mechanical and Materials Engineering, University of Denver, 2155 East Wesley Avenue, Denver, CO 80210, USA; spencer.brewster@du.edu (S.B.); paul.rullkoetter@du.edu (P.J.R.)

**Keywords:** humanoid robots, foot design, design optimization, anthropomorphic locomotion

## Abstract

This paper presents the foot design for humanoid robot Mithra, with the goal of biomimetically improving impact behavior, natural power cycling throughout the gait cycle, and balance. For this purpose, an optimization framework was built which evaluates the human-inspired objectives using a dynamic finite element analysis validated by benchtop experiments. Using this framework and through several concept design iterations, a low-cost, compliant foot was optimized, designed, and fabricated. The analyses showed that the optimized foot significantly outperformed the baseline rigid foot in approaching the characteristics of human feet. The proposed framework is not limited to humanoids and can also be applied to the foot design for lower-limb prostheses and exoskeletons.

## 1. Introduction

Advancements in materials, energy resources, and control methods are bringing humanoid robots closer to operating effectively in real-world environments. Despite significant progress in robot design, robotic feet still have a long way to go before they can fully replicate the complexity of the human foot. One of the greatest challenges and constraints in successful performance of a humanoid robot is keeping the robot’s balance and preventing falls. This is often quantified through a fundamental concept in legged robots, i.e., the zero-moment point (ZMP) [1]. The ZMP is the point on the ground where the combined moment of inertial and gravitational forces equals zero [1,2]. If the projection of this point falls within the support polygon, the robot is stable and can prevent itself from falling. This has inspired design of humanoid robots with large flat feet, allowing for a larger area in which the robot can maintain balance, even in the presence of uncertainties and controller errors [2]. Classic examples of such robots are ASIMO [3], WALK-MAN [4], and the HRP series humanoids [5,6,7]. However, this approach to foot design largely overlooks other important functions, including impact behavior and energy cycling.

From a different perspective, researchers have explored legged robots with compact, highly agile feet that feature reduced inertia and lack ankle actuators. The rationale behind this shift is that if a robot can swing its leg quickly enough, it can maintain balance by continuously adjusting the feet position [8]. The quintessential example of a robot with a point foot is Raibert’s hopper which achieves balance by rapidly shifting its single foot’s position while hopping [9]. The bipedal robots MABEL and ATRIAS implemented this design to reach stable and efficient walking and running [10,11,12,13].The bipedal robot Cassie also adopted this same approach for stability and mobility [14], even though its newer version, Digit, reverted to a large, flat foot design, similar to traditional humanoid robots [15].

While the previously mentioned robotic foot designs have prioritized stability and control in bipedal robots, efforts have also been made to develop feet that replicate other functional aspects. Recognizing the role of foot dynamics in locomotion, some works have investigated compliant feet and shock-absorbing mechanisms to minimize impact forces and enhance gait efficiency [16,17]. As an example, the humanoid robot LOLA features a uniquely designed foot that mimics the structure and function of the human foot [18]. This design separates the heel and the forefoot from the main structure, enabling independent flexion and extension of the toe and heel regions, while the heel includes a hydraulic damper and viscoelastic layers to reduce impact forces. The forefoot features an actuated toe designed to enhance stance stability, extend step length, and improve forward propulsion [18]. A notable drawback of such intricate foot design is the added weight, resistance, and need for actuation, which make control more challenging and decrease energy efficiency. After assessment, the Lola designers determined that a passive toe joint is more beneficial than an active one, unless a suitably light mechanism can be created [18].

More recently, in [19], it was aimed to achieve a similar objective with a three-part segmented foot with a foot shape inspired by human anatomy. Preliminary tests of this foot indicate that it can replicate human-like ground reaction forces during a simulated gait cycle [19]. In another work, a group of researchers developed a fully compliant foot composed of multiple interconnected links, aimed at enabling the foot to adapt to uneven terrain or small obstacles by adjusting its shape [20]. However, similar to the classic large robot feet, this design focused mainly on robustness and the ability to stand on uneven surfaces, without addressing foot power dynamics or impact absorption.

In contrast with legged robots, one field that particularly focuses on impact absorption and foot energy dynamics is the design of prosthetic feet. Prosthetic feet have aimed to mimic the human foot–ankle structure to help restore a natural gait and balance for users. The classic Solid Ankle Cushioned Heel (SACH) mimics the foot–ankle complex by a compliant heel that mitigates impacts through a cushion and a wedge that simulates plantarflexion [21]. The SACH foot is durable and cost-effective, but its power characteristics are very different from those of an able-bodied person, especially in the second half of the gait cycle. To address the power output limitations of the SACH foot, most of the more recent foot designs have transitioned to Energy Storage and Return (ESR) feet [22]. ESR feet are constructed from flexible materials that compress upon heel-strike to store energy. As the user moves through the gait cycle, this stored energy is released, functioning like a spring to generate power and assist with forward movement. Research has shown that ESR feet offer greater comfort during dynamic movements and enhance users’ ability to participate in such activities [23].

To bridge the gap between prosthetic feet and human feet, numerous studies have been conducted to optimize various design attributes. One such attribute is the geometrical path, known as the rollover shape, which represents the trajectory of the center of pressure (COP) during the gait cycle. Studies have found that rollover shapes are consistent among people with similar leg lengths and remain relatively constant despite changes in walking speed, heel height, or torso weight [24]. However, it has been suggested that rollover shape alone is inadequate as a design criterion for optimizing gait, as two prosthetic feet can share the same rollover shape but exhibit vastly different lower leg kinematics. As a solution, a new parameter called Lower Leg Trajectory Error (LLTE) has been introduced, which measures the error between the desired lower leg position throughout the gait cycle and the actual positioning of the limb equipped with a prosthetic foot [25]. From a different perspective, instead of focusing on the overall foot design, some studies have used numerical methods like finite element analysis (FEA) to refine specific aspects of prosthetic feet [26,27,28]. These studies are typically conducted with the primary aim of minimizing metabolic energy expenditure and reducing joint impact forces [29,30]. For example, using this idea, it was found that lowering ankle and heel stiffness while increasing the stiffness of the toe and mid-foot resulted in reduced metabolic cost and maintained natural knee loading [30].

While the rich body of literature for design of prosthetic feet provides valuable insights for design of a robot foot, we note that almost all of these designs and analyses have been conducted for feet accompanying passive ankles. This is mainly because powered prosthetic legs are a relatively recent innovation, and the field is still in its early stages of development [31,32,33,34]. An intriguing example is the powered prosthetic ankle–foot device SPARKy, where the designers utilized a commercially available ESR foot and modified the stiffness of the ankle–foot complex by motorizing a spring attached to the top of the foot [35]. This approach provided the ability to dynamically adjust the foot’s stiffness and generate ankle torque with minimal energy expenditure.

Based on this, the present work aims to close this gap in foot design for humanoid robots. In particular, we investigate how a low-cost, passive foot can be designed such that in connection with powered joints of an anthropomorphic humanoid robot, it approaches the power characteristics of human locomotion. The humanoid robot foot is developed for Mithra, which has been specifically designed and built to match the kinematic and kinetic attributes of an average adult human [36,37,38]. Since the feet are the primary drivers of the ground contact process, design of a foot that absorbs and releases power the way humans do is essential to achieving the goal of the anthropomorphic locomotion. By combining and extending design approaches from prosthetic and robotic feet, this research aims to develop a method for designing passive feet for humanoid robots, with potential applications in powered prosthetic legs.

Building on the research work discussing foot dynamics and prosthetic and robotic foot design, and combining the findings of each group, this paper presents the design of a novel robotic foot. While prosthetic foot designs are inevitably affected by the (passive) ankle side, our approach focuses exclusively on the distal foot’s performance and its specific potential to improve gait dynamics. In this way, inspired by the proposed design methodologies for prosthetic feet, optimization of the design will be carried out through a dynamic FEA model. Instead of investigating roll-over shapes or metabolic costs which require models too complicated for FEA analyses, our design method will focus on directly emulating distal foot power curves and reducing running impact forces. This approach will allow us to directly focus on the parameters of interest: natural gait, impact mitigation and balance, without confounding variables affecting results.

## 2. Methods

### 2.1. The Stiff Foot

**Figure 1:**
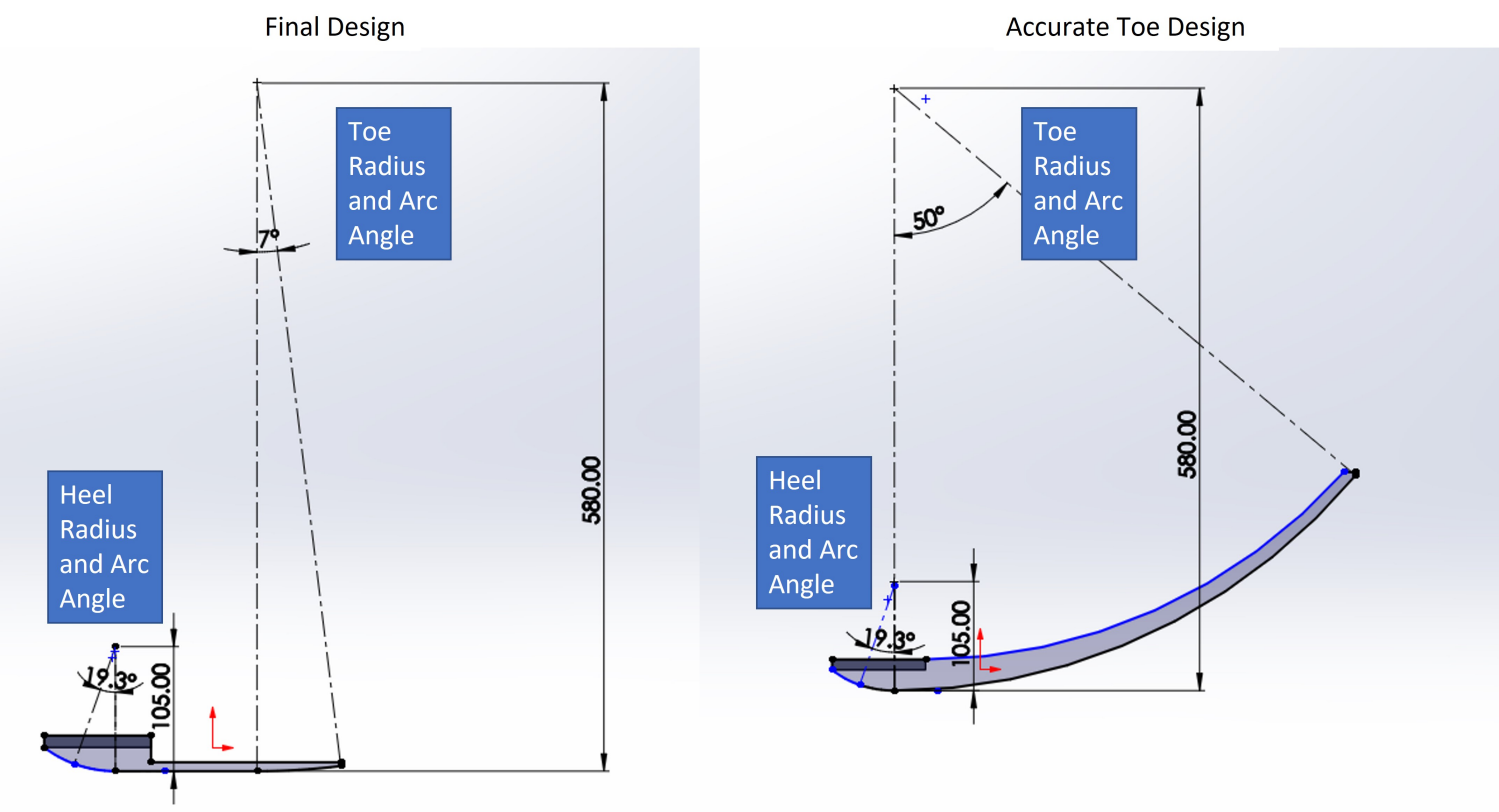
Sketch of the stiff foot illustrating the heel and toe radii along with their respective travel angles. The left image shows the selected model, while the right depicts the version with the full toe travel, which produced a foot that was excessively long and tall for practical use.

As a baseline, we created a rigid rollover-shaped foot to serve as a reference for comparison with the optimized flexible design. The stiff-foot geometry was developed from human gait measurements, based on the premise that the foot behaves as a non-slipping rolling surface during both heel-strike and toe-off. The heel and toe angles and their radii were derived by relating center-of-pressure (COP) trajectories with foot rotation during heel-strike and toe-off using the equation x=rθ, with *x* being the anterior distance traveled by the COP and θ the rotation of the foot. Analysis using the HuMod database [39] provided foot rotation measurements for heel-strike, mid-stance, and toe-off phases. The heel was determined to have a radius of about 105 mm with 19.3° of motion from heel-strike to foot-flat, while the toe had a radius of roughly 583 mm with 50° of motion from mid-stance to toe-off. Replicating the toe-off arc angle proved challenging, primarily because the human foot achieves this rotation through bending at the toe joint. Constructing a stiff foot with equivalent travel would produce an impractically long and tall design, resulting in awkward proportions. To address this, the radius was preserved but the travel was reduced to 7°, ensuring the top of the toe region remained flat while keeping the foot’s length and weight within design constraints. Figure 1 presents a 2D schematic of the foot with the radii and arc angles clearly marked, alongside a comparison between the two design alternatives.

**Figure 2:**
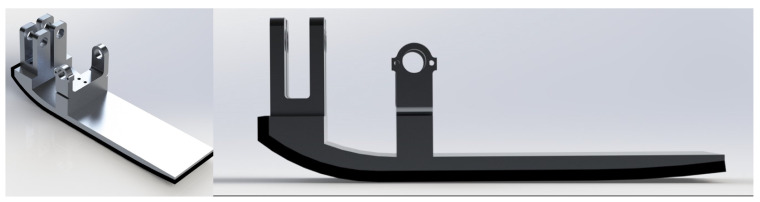
The CAD model of the stiff foot with curved heel and toe regions shown.

The middle region of the foot was set to be flat to facilitate stable standing. Alongside the rollover shape, the foot’s height and mass distribution were configured to position the ankle correctly for an individual matching the robot’s height. Finite element analyses confirmed the foot’s ability to endure gait cycle forces, while the foot’s design was refined to minimize weight. This stiff foot was machined out of Al 7075 to ensure strength and lightness, without the accompanying deformation and spring-like behaviors of most anthropomorphic feet. Figure 2 displays the finalized foot design, featuring universal connectors for the ankle joint and attached foot padding.

### 2.2. Design Objectives

#### 2.2.1. Foot Power

As mentioned, the main objective of the foot design in this work is achieving power characteristics similar to those of humans, and especially, the power absorption during the final stage of the stance phase which aids in push-off propulsion, thereby enhancing performance and efficiency [40,41]. Although a powered foot offers greater flexibility in replicating human-like power characteristics, as observed in [18], it comes at the cost of increased mass and energy consumption, which undermines the goal of improved efficiency. As such, in the present work, we focus on passive compliant feet and try to design their mechanics to approach the characteristics of human feet. Based on this, the first objective for our optimization framework was picked as the integral (sum in discretized form) of the squared error between the foot power obtained from the model and the reference human foot power during a gait cycle.

Evaluation of the gait cycle and the resulting power curve of the foot was based on the human foot power computation by Takahashi et al. [40] and prosthetic foot power analysis by Zhao [42]. A limitation of the approach in [40] is its dependence on conventional rigid body mechanics to calculate the foot’s angular velocity, despite the foot’s inherently deformable and elastic characteristics.This challenge is tackled by the deformable link segment (DLS) model as proposed in [42]. The DLS model simplifies the deformable area by representing it as a spring-damper system linking the foot’s COP to its center of mass (COM). Leveraging the FE model’s ready access to all parameters required by the DLS model and its existing 2D framework, foot power was computed using the DLS approach, with method of [40] serving as a benchmark for the desired distal foot power. A second-order low-pass Butterworth filter with a 100 Hz cutoff frequency was applied to the FE model’s calculated powers to remove noise caused by rounding errors or model inconsistencies. For the models with the best results, the filter’s effect was minimal.

#### 2.2.2. Impact Force

In addition to the foot power, we used a second objective for the design to assess the impact mitigation behavior of the feet, which is especially important in running. This objective was quantified as the peak force transferred to the knee. It is expected that the compliance of the foot absorbs some of the impulsive forces of the impacts, especially during running.

#### 2.2.3. Balance

In addition to gait dynamics, the feet are crucial for maintaining bipedal balance. To investigate the foot’s role in balance, Humphrey and Hemami developed a computational body model with a Head–Arm–Trunk (HAT) segment and represented the foot as a four-link structure [43]. Springs were added between the foot’s joints and at the ankle, and the study calculated the maximum forward lean achievable with different spring stiffness values before heel lift-off occurred, marking the loss of balance. They found that maximum lean angles varied from −4° (leaning back on the heels) to 11.75° (leaning forward over the toes) when the toe joint functioned as a spring, and ranged from −4° to 8.59° without toe joint actuation [43].

For this objective, we created an FE balance model to replicate the conditions and findings described in this study. The model consisted of a rigid HAT segment connected to the ankle actuator and foot. To assess the robot’s balance range for a given foot design, the HAT and ankle actuator were rotated by a specified angle and allowed to settle, enabling the foot to reach its natural standing deformation. Next, the HAT was released under gravity acting at its center of mass, accompanied by an ankle actuator force modeled as a torsional spring with stiffness derived from the computational study, aiming to restore the HAT to an upright posture. This procedure was repeated across various angles, with the balance limit identified as the point when the foot’s heel lifted off the ground.

#### 2.2.4. Total Objective

The total objective function used in the optimization process was formulated as the weighted sum of the above objectives. Moreover, we found that giving different weights to the foot power error objectives in the first and second halves of stance provides better results in terms of following the human foot power profile.

### 2.3. Foot Concepts

**Figure 3:**
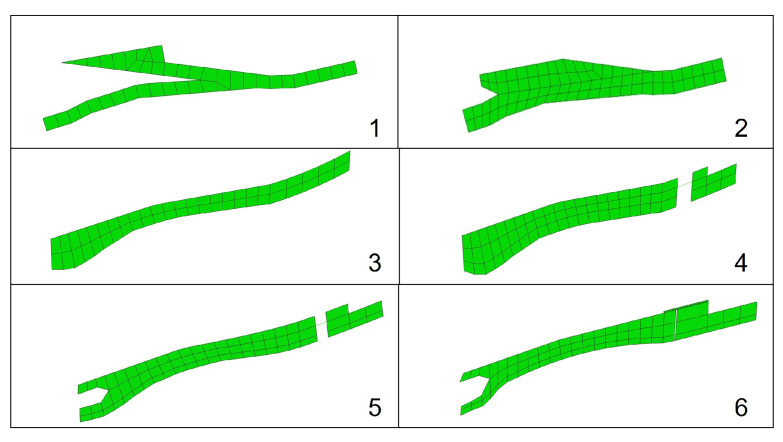
The evolution of the foot design as it adapted to meet various requirements. Foot 1 represented the original concept, with its form closely aligned to the computational model’s design principles. Foot 2 refined the shape further and aimed to minimize excessive deformation. Foot 3 enabled controlled flexing and lowered the ankle position. Foot 4 incorporated cushioning and introduced a toe spring. Foot 5 featured a softened heel, while Foot 6 transitioned to using a leaf spring at the toe.

During the design process, the foot underwent multiple conceptual variations before settling into a final concept, which was subsequently used for the optimization. Figure 3 shows a few of the foot iterations that we investigated.

Early prototypes sought to replicate the characteristics of ESR prosthetic feet but without incorporating an ankle segment. It quickly became clear that the ankle mechanism in Mithra directed forces toward the rear portion of the upper links, leading to excessive deformations. The ankle connection was subsequently lowered and integrated into the foot to reduce deformation, but this configuration proved overly bulky, restricting the foot’s ability to flex and absorb energy while also positioning the ankle joint higher than that of a human. The upper region was then further consolidated into the shape of the foot and a rounded heel was added to allow the foot to roll into stance phase and maintain contact with the ground. To enhance the foot’s power delivery, a toe joint equipped with a torsional spring was introduced, and a thin layer of impact-absorbing foam was applied to the sole, helping to dampen impacts and produce smoother ground reaction force profiles. It was later determined that a softer heel section was necessary for better impact absorption, prompting the rear portion of the foot to be split once more. The low-profile design was retained, while a narrower gap limited the ankle mechanism’s influence, preventing excessive heel deformation. Finally, when it became clear that a torsional spring with the required characteristics was impractical, it was replaced with a carbon fiber leaf spring. The leaf spring was mounted to the top of the foot, enabling rotational movement while restoring the foot to its neutral position after deflection.

### 2.4. Finite Element Model

#### 2.4.1. Joint Moment Modeling

Developing a precise finite element model depended greatly on comprehending the human gait cycle and how various input torques combine to generate the intended movement. In a sequence of studies, Shamaei et al. investigated the quasi-stiffness characteristics of lower-limb joint moments [44,45,46]. They segmented the human gait cycle into five distinct sub-phases, each characterized by unique stiffness values. From these findings, they created generalized equations for each of the joints during both flexion and extension based on the height and weight of the individual. Since the leg joints of Mithra are to be controlled using human-like impedance control based on such impedance values [37,38], we use this method for determining the leg joint moments during the gait cycle.

**Figure 4:**
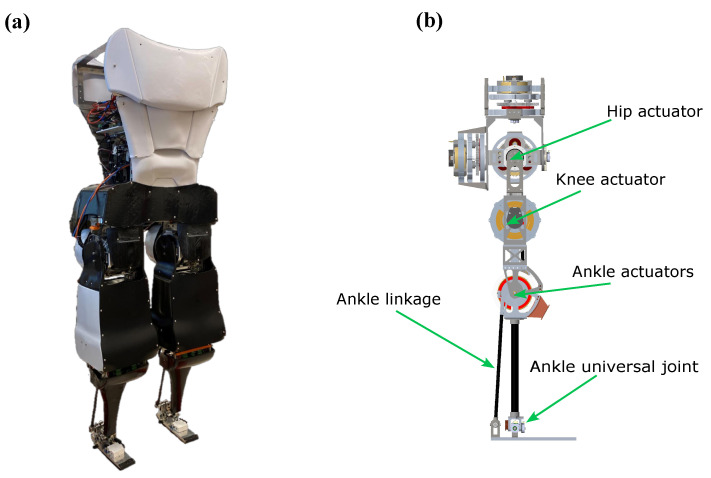
(**a**) Mithra, the humanoid robot the feet were designed for. (**b**) The leg mechanism of Mithra and its actuators as used in our analyses.

Similarly, another research team examined toe joint stiffness across the gait cycle to gain insights for designing a more effective prosthetic toe [47]. Because Mithra features a passive toe joint to minimize weight and inertia at the leg’s extremity, the spring’s reference angle and stiffness were adjusted according to their findings and incorporated as optimization variables when evaluating foot power and impact forces. Figure 4 displays a labeled CAD model of Mithra’s leg, serving as a reference for the upcoming discussion on the finite element model.

#### 2.4.2. Modeling the Robot

**Figure 5:**
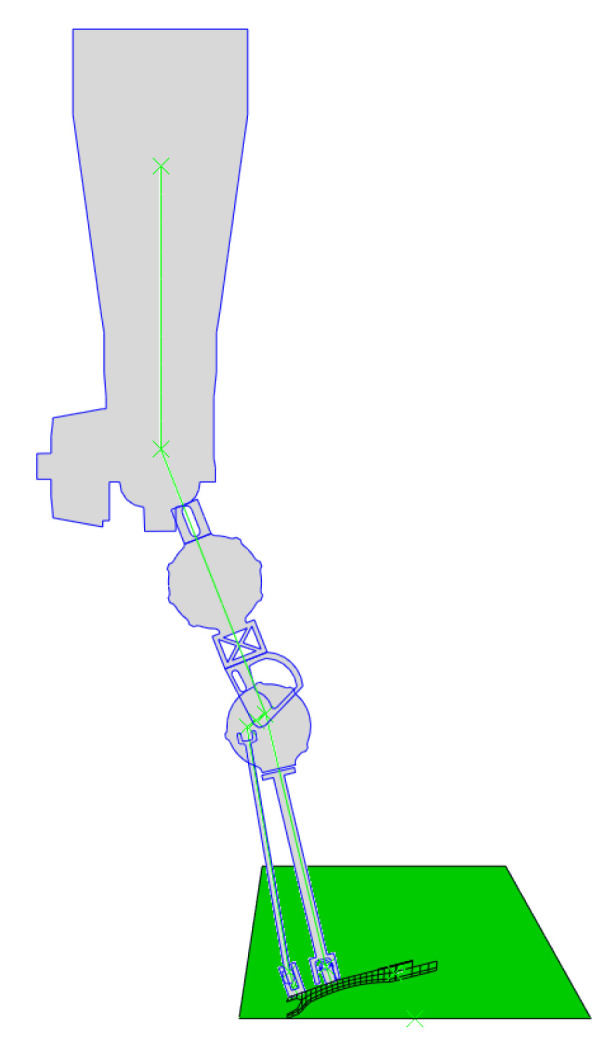
The FE model setup, with joints positioned at angles corresponding to the heel-strike phase of the gait cycle.

The finite element model is developed as a 2D sagittal plane representation, featuring a deformable foot linked to rigid components that comprise the ankle setup, shank, thigh, and torso of the robot (Figure 5). The foot is attached to the shank and rear ankle linkage via pin joints connected to universal connectors, which are rigidly fixed to the foot’s top flat surface. Pin joints connect the shank to the thigh, the ankle setup, and the universal ankle joint. Likewise, the thigh is linked to both the shank and torso using pin joints. Rotational spring connectors are installed at each joint to simulate the robot’s impedance-controlled motors. The joint springs generate torque based on the angular displacement between the connected bodies.

Mithra’s ankle joints are powered via a spatial linkage system with motors placed at the knees. Therefore, in the FE model, ankle torque is transmitted through the planetary carrier connector linkage to the foot, with a reaction force applied to the shank. For more details of Mithra’s ankle mechanism, see [38,48].

Mass distribution, height, and link lengths were specified according to the robot’s real dimensions, generally aligning with the positions of the depicted components in Figure 5. Gravity was modeled as an external force, and a hard contact interaction was established between the foot and the ground, incorporating a damping coefficient of 0.01 and a friction coefficient of 0.75. Initial models used a simplified friction approach that prevented slipping, but this led to excessive forces in some components during toe-off. In models featuring a distinct toe segment, contact between the toe and the main foot was omitted, as the physical design includes curved areas permitting free rotation between these sections. Likewise, contact was not specified between the leaf spring and the toe’s upper surface as it was found not to show a significant effect and thus was omitted to speed up the optimization process.

Alongside gravity, external forces were applied at the hip to simulate the presence of the opposite leg and foot, which is important during the double stance phases at the start and end of the gait cycle. Hip reaction forces were calculated using Newton–Euler techniques assuming rigid links using data from the HuMod database [39], with reaction forces being translated from the foot through the ankle to the shank and ultimately to the thigh and hip. The running simulation was configured similarly to the walking model, with joint angles, stiffness values, reaction forces, step durations, and initial velocities adjusted for the running conditions.

#### 2.4.3. Element Selection and Materials

The system was modeled in Abaqus 2020 HF1 (Dassault Systèmes, Vélizy-Villacoublay, France) using the CPS4R element, a 4-node bilinear plane stress quadrilateral featuring reduced integration and hourglass control. This element was chosen for its suitability in explicit analyses and its superior in-plane bending performance. Reduced integration helps the element avoid shear locking, while hourglass control enables a relatively coarse mesh to accurately capture the model’s behavior. Additionally, this element facilitated easy manipulation within MATLAB R2022a (Mathowrks, Natick, MA, USA), aiding in geometric optimization tasks.

The foot was modeled using three different types of materials, nylon 6/6, carbon fiber, and aluminum. Nylon has been utilized in previous prosthetic foot research [24,29,49] and It was selected as the primary material for the foot due to its ease of manufacturing, low cost, high flexural strength, and elastic characteristics. An impact-absorbing foam with hyperelastic properties, modeled after commercially available D3O^®^ foam (D3O, Croydon, UK), was incorporated on the sole of the foot [50].

### 2.5. Optimization Variables

**Figure 6:**
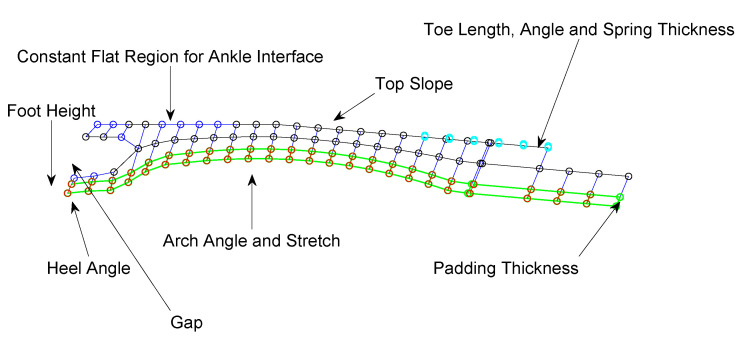
An annotated diagram of the foot showing the parameters adjusted to generate shape variations for optimizing the foot geometry.

Once the general foot shape was set and the FE model was created, optimization was performed through changing foot parameters in MATLAB to create unique and viable foot shapes. Figure 6 illustrates the various parameters that were modified to identify the optimal foot design. Foot height controls the vertical thickness of the design and plays a key role in ensuring the structure can withstand applied forces. As reported in [51], the range of the ankle joint center height in humans is between 65.3 mm and 78.2 mm. In Mithra, the height of the ankle joint center with respect to the top of the foot is 30 mm. Additionally, each foot includes a 6 mm layer of impact insulation material and force sensors at the bottom. Based on these measurements, we restricted the foot height to between 30 and 40 mm to remain within the anthropomorphic range.

The heel angle, together with the gap size, influenced the rollover shape and the foot’s power profile during the heel-strike phase of the gait cycle. Gap size defines the separation between the top and bottom surfaces of the heel region and thereby affects the heel’s thickness. The arch angle governed a set of constrained angles that maintained a level foot while influencing its deformation and spring-like behavior throughout the gait cycle. The arch stretch parameter lengthened the foot and shifted the top of the arch relative to the constant section. This parameter was crucial for keeping the mesh square as the foot shape evolved and for enhancing impact absorption when the forefoot made ground contact. The top slope parameter enables a smooth transition from the constant region to the toe area, determining the forefoot’s thickness and influencing its deformation from mid-stance through toe-off. Finally, the toe parameters of length, angle, and spring thickness were adjusted to control how the toe interacted with the ground.

### 2.6. Optimization Framework

**Figure 7:**
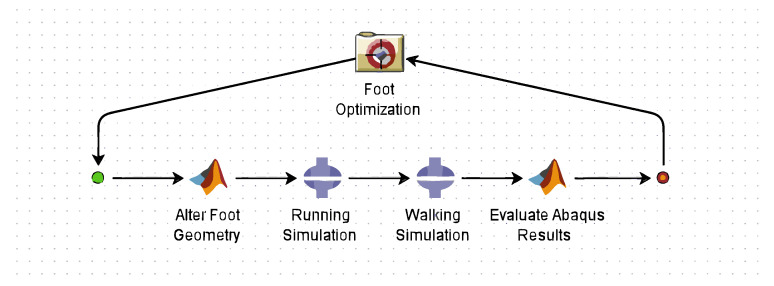
A schematic showing the optimization framework loop using Abaqus, MATLAB, and Isight.

Optimization was carried out in the Isight design platform (version 5.9, Dassault Systèmes, Vélizy-Villacoublay, France), which allowed seamless integration of all components into a unified optimization algorithm. As shown in Figure 7, the Isight software enables multiple programs to interface and exchange data seamlessly. In this framework, the optimization algorithm selects new parameters, MATLAB generates the foot geometry, Abaqus executes the FE model and sends the results back to MATLAB, where the outputs are evaluated and the algorithm updates the parameters based on the objective evaluations. This process was carried out separately for each FE simulation (walking, running, and balance), each evaluated according to its specific design objectives: foot power, impact mitigation, and lean angle.

The Isight toolkit offers a diverse range of optimization techniques to address a broad spectrum of problem types. Several of these algorithms were explored and evaluated for their effectiveness. Given the highly nonlinear nature and lengthy runtime of the simulations, only a select few algorithms capable of handling such challenges will be discussed. Ultimately, the Downhill Simplex method [52] was employed to identify an initial search region, followed by the Pointer method [53] to refine and obtain the final optimization results.

### 2.7. Physical Tests

The physical testing of the fabricated foot was performed using an Instron Universal Testing System (Instron, Norwood, MA, USA). The tests adhered to material testing protocols established in several prosthetic foot studies [24,26,27]. The test setup is straightforward: the foot is secured at its ankle connection to various fixtures within the Instron machine beneath the force applicator, allowing evaluation in multiple positions. A foot-sized platform, acting as the ground, is attached to the force applicator. FE models of these tests will also be created for comparison and to develop force profiles that maintain stresses within a specified safety factor. Although this approach does not simulate impact events, it offers a solid foundation for assessing the accuracy of the FE model. Careful attention is required to ensure that the recorded results reflect foot deformation rather than any movement or compliance in the fixturing. To achieve this, stainless steel components and sturdy support fixtures were employed.

## 3. Results

### 3.1. Stiff Foot Analysis

**Figure 8:**
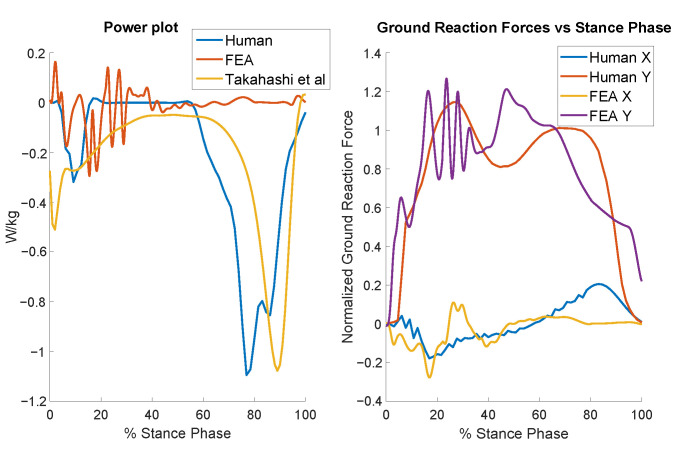
The stiff foot results for power and ground reaction forces. The foot performs well at maintaining low ground reaction forces but falls short in power absorption.

As expected, the rigid rollover-shape-based foot struggled to accurately replicate foot power generation throughout the gait cycle. Figure 8 presents the stiff foot’s results, highlighting the points discussed. While it maintained low ground reaction forces, the foot struggled to settle once it reached the flat position. Regarding power absorption, the initial contact phase shows some negative power, but the data is noisy and does not align properly with the timing observed in human gait. At toe-off, there is nearly no power absorption, which is expected due to the absence of a flexible parts and the restricted toe arc angle in the foot design. Furthermore, the decline in ground reaction forces near the cycle’s end supports the notion that deformable parts and their associated power absorption are crucial for extending push-off duration and enhancing forward propulsion.

**Figure 9:**
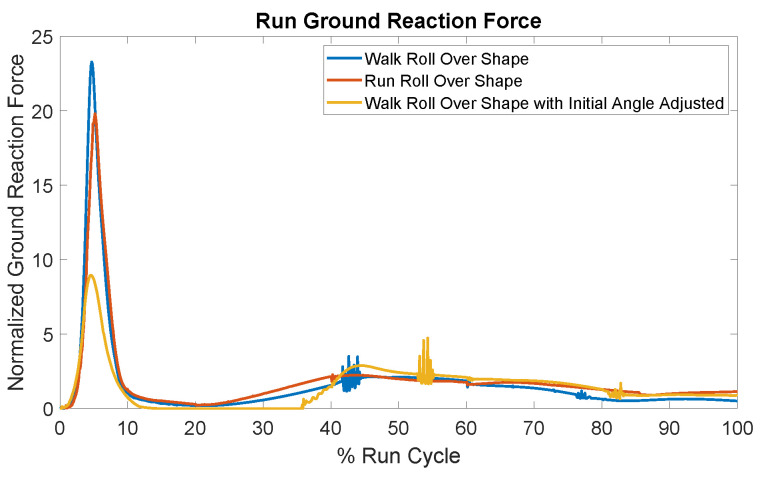
Ground reaction forces for running with the stiff foot. The foot’s inability to absorb impact leads to a large force spike upon ground contact. Adjusting the foot’s rollover shape to match running data yields only a minor improvement. However, modifying the ankle angle to align with the walking rollover angle results in a substantial reduction in forces.

The drawbacks of a stiff foot is more prominent in the case of running. The ground reaction forces for this case are presented in Figure 9 where without adequate impact absorption, the foot bounces off the ground, causing a significant spike in force. One possible reason was that the stiff foot excelled at minimizing impact forces during walking because it was specifically designed for that purpose, but lacked the adaptability needed to perform well in other tasks. To investigate this hypothesis, a second stiff foot model was developed using running data to shape the rollover curves for the heel and toe regions. The outcomes of this test, also displayed in Figure 9, show a modest improvement, but the forces still remain excessively high. We further hypothesized that the stiff feet struggled with running because, unlike walking where the foot rolls off the heel, running typically involves landing almost flat-footed or even on the forefoot. This insight prompted us to adjust the foot’s initial angle during the running cycle to better align with walking data and the results can be seen in Figure 9. The forces were notably reduced, supporting the validity of our second hypothesis.

### 3.2. Compliant Foot Analysis

**Figure 10:**
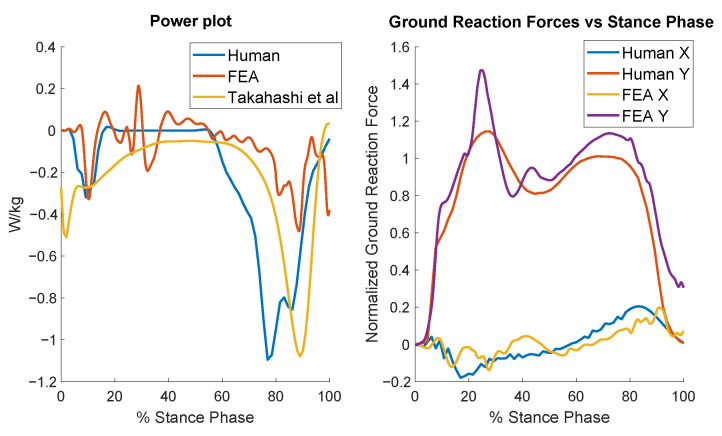
Results for the distal foot power (**left**) and ground reaction forces (**right**) across the walking gait cycle for the optimized compliant foot.

Figure 10 presents the results for the optimally designed compliant foot. At the start of the cycle (after impact), the distinctive negative power dip appears as the heel deforms and the foot nears ground contact. Some foot geometries improved impact absorption, but they either could not endure the stresses of running or limited the foot’s ability to absorb power during the push-off phase. After the impact settling, the power remains relatively low until the terminal stance and push-off phases, where it shows improvement but still falls short of the negative value observed in human data. Figure 10 also displays the normalized ground reaction forces for both the model and human data. The model exhibits the characteristic bimodal peaks. Interestingly, the force magnitudes of the model are close to those of humans, even though the foot was not optimized for matching the force magnitudes.

**Figure 11:**
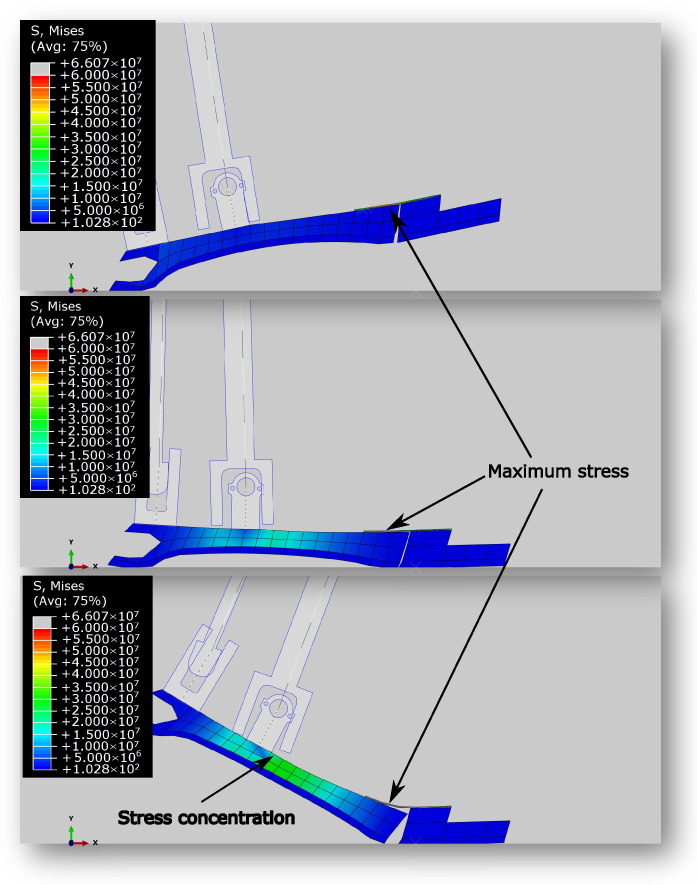
The stress contours of the foot during various points of the walking gait cycle. (**Top**): Initial Impact. (**Middle**): Toe Impact. (**Bottom**): Toe-Off. The maximum stress always occurs in the spring connecting the toe to the foot. During push-off, the stress of the points close to the ankle universal joint connection increase due to stress concentration.

Figure 11 presents stress contours of the foot at various key moments throughout the gait cycle. Note that the area of maximum stress occurs where the foot begins to slope away from the rigid connector section.

**Figure 12:**
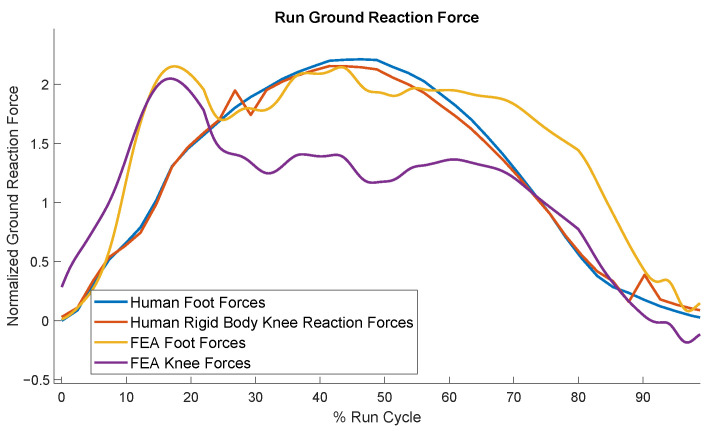
Comparison of the model’s running force profiles with those of humans.

Figure 12 shows the comparison of the resulted running forces of the model with those of humans. As expected, the impact forces in the model are greater than those in humans, but notably, the optimized foot design has been able to keep the impact force close to the maximum running force. This becomes more remarkable when compared to the results of the stiff foot (Figure 9). The force profiles of the model and the human do not align closely, presumably because the optimization only targeted minimizing the impact forces for the running case. Similar to the walking outcomes, the running data exhibited trade-offs: some results better matched human data or reduced knee reaction forces, but considering foot stress constraints and the focus on optimizing walking power, this was the best compromise that could be achieved.

**Figure 13:**
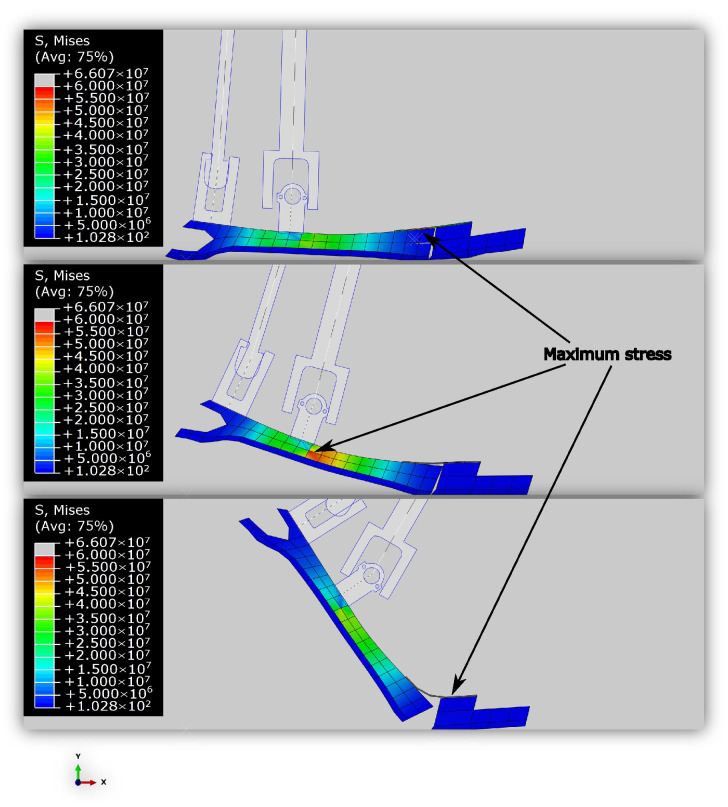
Stresses of the foot during the running gait cycle at various points. (**Top**): Initial Impact. (**Middle**): Maximum Stress. (**Bottom**): Toe-Off. Similar to walking, maximum stress during touchdown and push-off occur at the spring, but the stress concentration at the ankle universal joint connection in the middle of the stance phase is higher in this case.

Figure 13 illustrates the foot’s stress distribution throughout the running cycle, showcasing the FE model in operation. Again, note the area of maximum stress occurs where the foot begins to slope away from the rigid connector section.

**Figure 14:**
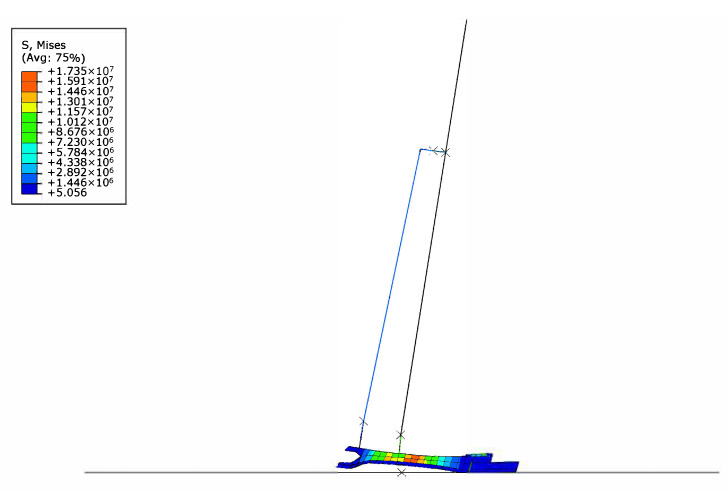
The balance model simplified with a HAT portion connected to the ankle. The model is then rotated through different angles to identify the maximum achievable lean angle.

In contrast to the other two optimization objectives, altering the foot’s geometry had little effect on the model’s maximum achievable lean angles. Most designs exhibited forward lean limits between approximately 3° and 5°, and backward lean limits between 2° and 4°, before the heel or toe lifted off the ground. A few designs achieved up to 6° of lean, but these feet were heavy and underperformed in both running and walking simulations. The final design featured a forward lean limit of 5° and a backward limit of 3°. This result fell short compared to humans, who achieve lean angles around 8.58°, presumably because the human foot functions as a multibody system. Figure 14 shows an illustration of the balance analysis using FE.

**Figure 15:**
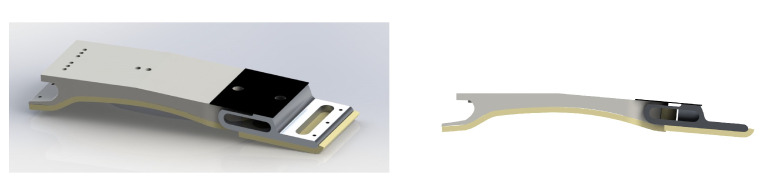
The final model of the foot.

Based on the optimal results presented, the compliant foot was designed and built as shown Figure 15. The overall dimensions of each foot are 274×75×33 mm with a weight of 325 g.

### 3.3. Physical Test Results

**Figure 16:**
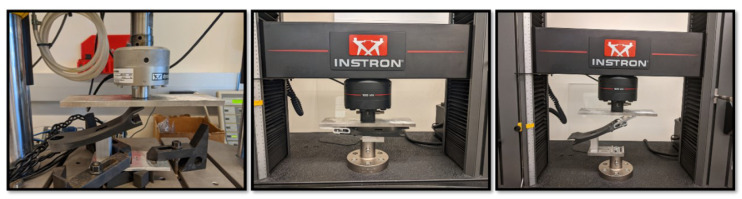
The physical testing setup. (**Left**): the heel test, (**Center**): the mid-foot test, (**Right**): the toe test. Stiffness values were assessed by tracking loads and displacements.

**Figure 17:**
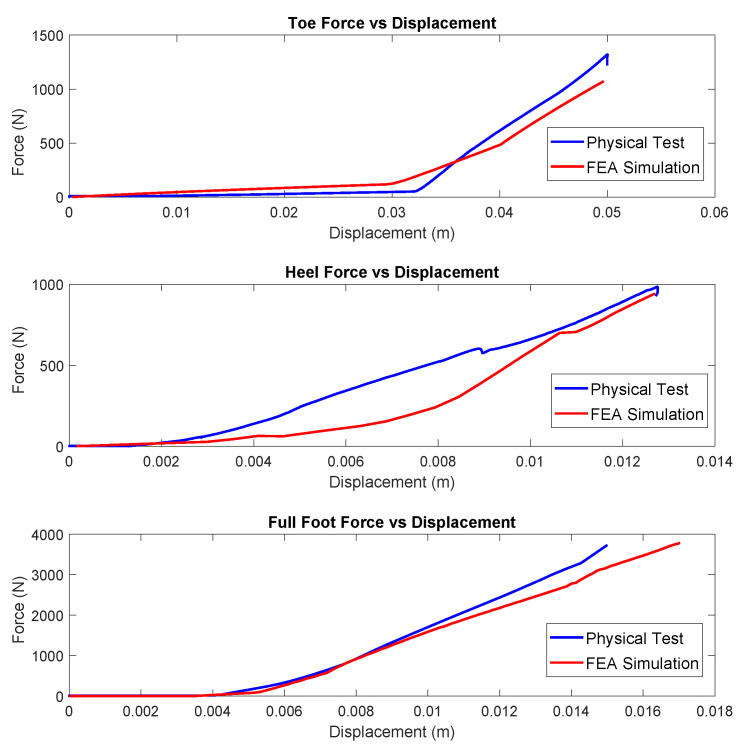
Comparison of the physical test results with the FEA simulations. The greatest discrepancy lies in the toe test, but across the spectrum, the results are close.

Figure 16 shows the physical test setup for different angles. Although physical testing highlighted certain limitations of the FE model in capturing subtle foot behaviors, the results were sufficiently close to validate the model’s overall accuracy. As can be seen in Figure 17, the greatest error occurred in the assessment of the toe region. This discrepancy can be attributed to the challenges in accurately modeling the connection between the foot and the leaf spring, as well as the significant moment experienced by this section of the model. In the model, the leaf spring exhibits greater stiffness than observed in physical tests, while the forefoot appears more compliant. Together, these differences result in an overall toe assembly error of 11.41% across the entire structure.

## 4. Discussion

With the physical testing results validating the FE model, meaningful conclusions can be drawn regarding the foot design and the overall findings of this research. The optimized compliant foot design can, to a good extent, follow human walking impact power absorption profiles and their push-off power characteristics. Similarly, this design achieved running impact forces comparable in magnitude to those of humans. The study, however, found that altering foot geometry had negligible effects on balance outcomes. This is likely due to the lack of toe and forefoot actuation, as well as the multibody structure of the human foot, both of which help maintain complete ground contact. The advantages of the designed foot become more apparent when compared to the stiff feet commonly used in state-of-the-art humanoid robots. While a stiff foot designed to replicate the human COP roll-over shape was able to keep ground reaction forces low during walking gait cycle simulations, it exhibited minimal power absorption. Moreover, in the case of running, the resulting ground reaction forces were prohibitively high. Further exploration of this phenomenon suggested that it stems from the lack of adaptability in a foot designed for walking heel-strike conditions when applied to running. In contrast, one of the greatest strengths of the flexible foot lies in its ability to adapt to the varying conditions and demands the robot may encounter.

The study found that combining a flexible heel, a deformable mid-foot region, and a flexible toe yielded the best overall results. Throughout the design process, valuable insights were gained on how foot shape modifications could enhance the replication of human capabilities. One key lesson learned was the importance of segmenting the foot. Partially separating the heel from the rest of the foot by introducing a gap between the top of the foot and the bottom of the heel proved effective in increasing power absorption, reducing impact forces, and lowering stresses in critical regions of the foot. Likewise, incorporating a toe connected via a spring was instrumental in enabling the foot to begin achieving human-like push-off power absorption while also withstanding the stresses encountered during running. Although the final design exhibited qualitative power behavior similar to the human foot, the limitations of a passive foot, even with the addition of a spring, prevent achieving the exact power patterns observed in humans. Using variable-stiffness and/or damping systems, or small actuators can certainly improve this, but at the cost of added weight, as observed in [18]. Likewise, the smaller lean angle compared to humans implies that the actuators must contribute more to maintaining leaning stability, which can, in turn, impact energy efficiency. On the other hand, adding linkages (as in [43]) may increase foot weight and potentially result in additional energy expenditure as well.

The main trade-off in the optimization process was keeping the stresses within the material limits while achieving human-like power characteristics during impact and push-off. This was especially complex considering high running forces and constraints such as low cost which excluded the use of stronger materials such as customized carbon fiber parts. Heftier designs tended to reduce the foot’s power absorption capacity, whereas lighter designs were unable to withstand the running forces. As such, adding a toe-connecting spring and the flexible heel design were critical to resolve this problem. Likewise, in some earlier concepts (e.g., iteration 2 in Figure 3), it was observed that a trade-off exists between impact absorption and push-off power characteristics. However, incorporating a toe spring and a softer heel largely decoupled these objectives, which left the primary trade-off between maximum stress and the power absorption capacity of each part of the foot.

The greatest variations among the final models were observed in the foot height, as well as in the angle and length of the arch. These parameters can be adjusted in combination to produce feet with similar performance outcomes but differing shapes and sizes. This mid-foot region governs the overall deformation of the foot during the gait cycle, first compressing to absorb impact and then bending in coordination with the toe to generate push-off power absorption. Understanding the interaction between these parameters could enable the creation of feet tailored to specific design constraints, accommodating both larger and smaller robots.

## 5. Conclusions and Future Work

We developed an optimization framework for design of low-cost compliant feet for humanoid robots with biomimetic behavior. The framework is based on coupling the optimization in MATLAB with dynamic finite element analysis in Abaqus, which was validated through benchtop experiments. Multiple concept design iterations were carried out to achieve the most favorable outcomes for the defined objectives. The final optimized foot outperformed the stiff foot in every evaluated aspect. However, despite this superior performance, without actuation or variable damping, the designed foot still fell short of the human foot performance, especially during push-off. Incorporating these components would substantially increase the foot’s cost and/or weight, and was therefore deemed beyond the scope and objectives of this project. Importantly, note that the proposed framework is not limited to humanoid robot foot design as its biomimetic foundation also makes it applicable to designing feet for prostheses and exoskeletons, and particularly powered devices.

Future research will focus on testing the feet on Mithra and in real-world conditions. To support this, a human-inspired controller is being developed to enable evaluation of the foot’s biomimetic behavior. Once these tests are completed, a more definitive evaluation of the design can be made, guiding future development directions. Further, building on the insights from this study, other future directions may include designing a lightweight actuated toe and conducting a full 3D assessment of the gait cycle. The results of this study indicate that an actuated toe could be essential for attaining exact human-like balance and power profiles throughout the gait cycle. However, as noted in the prior literature, it must be designed so that its advantages outweigh the added inertia at the leg’s end and the increased complexity it introduces to control. Additionally, the current design is limited to sagittal plane analyses, since this plane captures the foot’s most prominent functions. A more comprehensive approach would involve modeling the gait cycle in 3D, accounting for movements and torques in the frontal plane.

## Data Availability

The raw data supporting the conclusions of this article will be made available by the authors on request.
